# Green copper oxide nanoparticles for lead, nickel, and cadmium removal from contaminated water

**DOI:** 10.1038/s41598-021-91093-7

**Published:** 2021-06-15

**Authors:** Alaa El Din Mahmoud, Khairia M. Al-Qahtani, Sahab O. Alflaij, Salma F. Al-Qahtani, Faten A. Alsamhan

**Affiliations:** 1 Environmental Sciences Department, Faculty of Science, Alexandria University, Alexandria, 21511 Egypt; 2 Green Technology Group, Faculty of Science, Alexandria University, Alexandria, 21511 Egypt; 3 Princess Nourah Bint Abdulrahman University, Riyadh, Saudi Arabia; 4 Center of Excellence for Advanced Materials and Manufacturing, King Abdulaziz City for Science and Technology, Riyadh, Saudi Arabia

**Keywords:** Environmental sciences, Nanoscience and technology

## Abstract

Environmentally friendly copper oxide nanoparticles (CuO NPs) were prepared with a green synthesis route without using hazardous chemicals. Hence, the extracts of mint leaves and orange peels were utilized as reducing agents to synthesize CuO NPs-1 and CuO NPs-2, respectively. The synthesized CuO NPs nanoparticles were characterized using scanning electron microscopy (SEM), Energy Dispersive X-ray Analysis (EDX), BET surface area, Ultraviolet–Visible spectroscopy (UV–Vis), and Fourier Transform Infrared Spectroscopy (FT-IR). Various parameters of batch experiments were considered for the removal of Pb(II), Ni(II), and Cd(II) using the CuO NPs such as nanosorbent dose, contact time, pH, and initial metal concentration. The maximum uptake capacity (q_m_) of both CuO NPs-1 and CuO NPs-2 followed the order of Pb(II) > Ni(II) > Cd(II). The optimum q_m_ of CuO NPs were 88.80, 54.90, and 15.60 mg g^−1^ for Pb(II), Ni(II), and Cd(II), respectively and occurred at sorbent dose of 0.33 g L^−1^ and pH of 6. Furthermore, isotherm and kinetic models were applied to fit the experimental data. Freundlich models (R^2^ > 0.97) and pseudo-second-order model (R^2^ > 0.96) were fitted well to the experimental data and the equilibrium of metal adsorption occurred within 60 min.

## Introduction

Nanotechnology has gained more attention since the synthesized materials are on the nanoscale that differ in chemical and physical properties from those of bulk materials. This allows the integration of nanomaterials in environmental^1,2^, medicinal^3^, and agricultural^4^ applications.

Several synthesis approaches have been used to produce metallic oxide nanoparticles, including physical and chemical routes. In literature, there are many reported techniques for the synthesis of Copper oxide nanoparticles (CuO NPs) via thermal reduction and microwave irradiation^5^, chemical vapor deposition^6^, polyol^7^, photochemical^8,9^, and electrochemical methods^10^. Most of these mentioned techniques have potential environmental impacts because they involve the use of  harsh, dangerous, and toxic chemicals in addition to being very expensive with costly reaction conditions. Akintelu et al.^11^ recommended that more research work should be conducted to minimize the toxicity of CuO NPs synthesis route while maintaining and/or improving their performance in environmental or medical applications. Therefore, green chemistry routes have attracted researchers’ interest for producing environmentally friendly metal nanoparticles that are free from the use of expensive, harsh, and toxic chemicals.

As there is a fast progress in the field of nanotechnology, the green synthesis of metallic oxide nanoparticles using plant extract has presented as an eco-friendly science by which there exists an ability to control the size, shape, and material quality^12,13^. The green synthesis technique is dependent on eco-friendly reducing and capping agents so it eliminates the generation of toxic intermediate during chemical reactions^14^. This would prompt researchers to develop non-toxic green synthesis methods for producing CuO NPs.

Water pollution is a global problem with the increasing usage of chemical compounds^15^. This can be due to the progress in industrialization and technological development, and the runoff of household wastes^16,17^. It is believed that heavy metal pollution is one of the serious factors affecting water bodies because of their toxic, non-biodegradable, and persistent nature when released into the environment through natural sources (weathering, erosion) and anthropogenic sources (car exhausts, industrial discharges, and mining)^18^.

The burgeoning demand for obtaining high-quality water has become a reason for researchers to develop advanced technology to deliver clean water. There are many conventional wastewater treatment techniques including precipitation, flocculation, electrocoagulation, ion exchange, etc. Ion exchangers are classified into organic and inorganic. However, composite ion exchangers are preferable to be applied in the removal of heavy metals because of their mechanical stability and enhanced ion exchange capacity which are lacked in organic or inorganic resins. For instance, Mohammad et al.^19^ found that the poly (3,4-ethylenedioxythiophene): polystyrene sulfonate-Zr(IV) phosphate enhanced the ion exchange capacity of Cd(II) to be 2.34 meq g^−1^. Whereas the composite of carbon nanotubes with cerium(IV) phosphate possesses Cd(II) exchange capacity of 1.64 meq g^−1^^20^. Polyvinyl alcohol Ce(IV) phosphate composite proved its efficiency as ion exchange for the mixture of Cu(II)–Zn(II), Cu(II)–Cd(II), and Cu(II)–Ni(II)^21^. However, the synthesis routes require many chemical reagents which harm the environment and most of the above-mentioned techniques cannot remove heavy metals from wastewater completely.

The most common adsorbents used in removal of a wide range of heavy metals are activated carbon and zeolite, but their costs are still high at large scale applications^22,23^. Accordingly, agricultural or plant byproducts can be an alternative for the preparation of adsorbents or nanosorbents. This has led to the integration of various nanomaterials in the adsorption process for the removal of heavy metals from water and/or wastewater.

Nanomaterials possess the potential of heavy metal removal from water over conventional techniques because of their high surface area (surface/volume ratio)^24^. As an example, metallic oxide nanoparticles can be used to provide a long-term solution to/for water quality and make possible water reuse^25^.

Plant extracts assisted CuO NPs have attracted much attentions because green synthesis techniques hold several advantages over chemical ones, which are using non-toxic solvents such as biological extracts in addition to their simplicity. For instance, aqueous black bean extract^26^, fruit extract of *Duranta erecta*^27^, *Eclipta prostrata* leaves extract^28^, *clove* extract^29^, *Solanum lycopersicum* leaf extract^30^, and Hawthorn berries extract^31^.

Herein, we focus on green preparation of CuO NPs due to the abundance of copper, its cost effective preparation, and its excellent optical, mechanical, thermal, electrical and catalytic properties^11,12^. Recently, CuO NPs have been made available for various applications. This led the researchers to apply alternative sustainable synthesis techniques. Singh et al.^32^ synthesized CuO NPs synthesized using *Psidium guajava* leaf extract as reducing agent as well as capping agent. They confirmed its potential for photocatalytic degradation of Nile blue (93%) and reactive yellow 160 (81%) dyes in 120 min. Khani et al.^33^ used the fruit extracts of *Ziziphus spina-christi* (L.) as reducing agents to prepare CuNPs and tested in crystal violet (CV) adsorption. CV removal reached 95% with a high adsorption capacity (37.5 mg g^−1^) in 7.5 min. Currently, CuO NPs were successfully synthesized using the seed extract of *Caesalpinia bonducella* but evaluated for electrochemical detection of riboflavin^34^.

Most literature are focused on the application of chemically synthesized CuO NPs in heavy metals removal from contaminated water. Fakhri^35^ prepared CuO NPs by sol–gel method and its uptake capacity for Hg(II) reached 46.10 mg g^−1^ at pH of 9 and nanosorbent dose of 0.05 g. Another CuO NPs were prepared by chemical precipitation technique and applied for Ni(II) removal from water^36^. Its adsorption capacity was 15.4 mg g^−1^ with nanosorbent dos of 0.2 g L^−1^, pH of 7.0, and contact time of 90 min.

Consequently, the objective of our work is to synthesize nontoxic CuO NPs by using the extracts of mint leaves and orange peels as green reducing agents. The obtained CuO NPs are tested as an adsorptive nanomaterial to purify polluted water from heavy metals such as Pb(II), Ni(II), and Cd(II) as well as modelling the experimental results with isotherm and kinetic models.

## Results and discussions

### Characterization of nanoparticles

**Figure 1:**
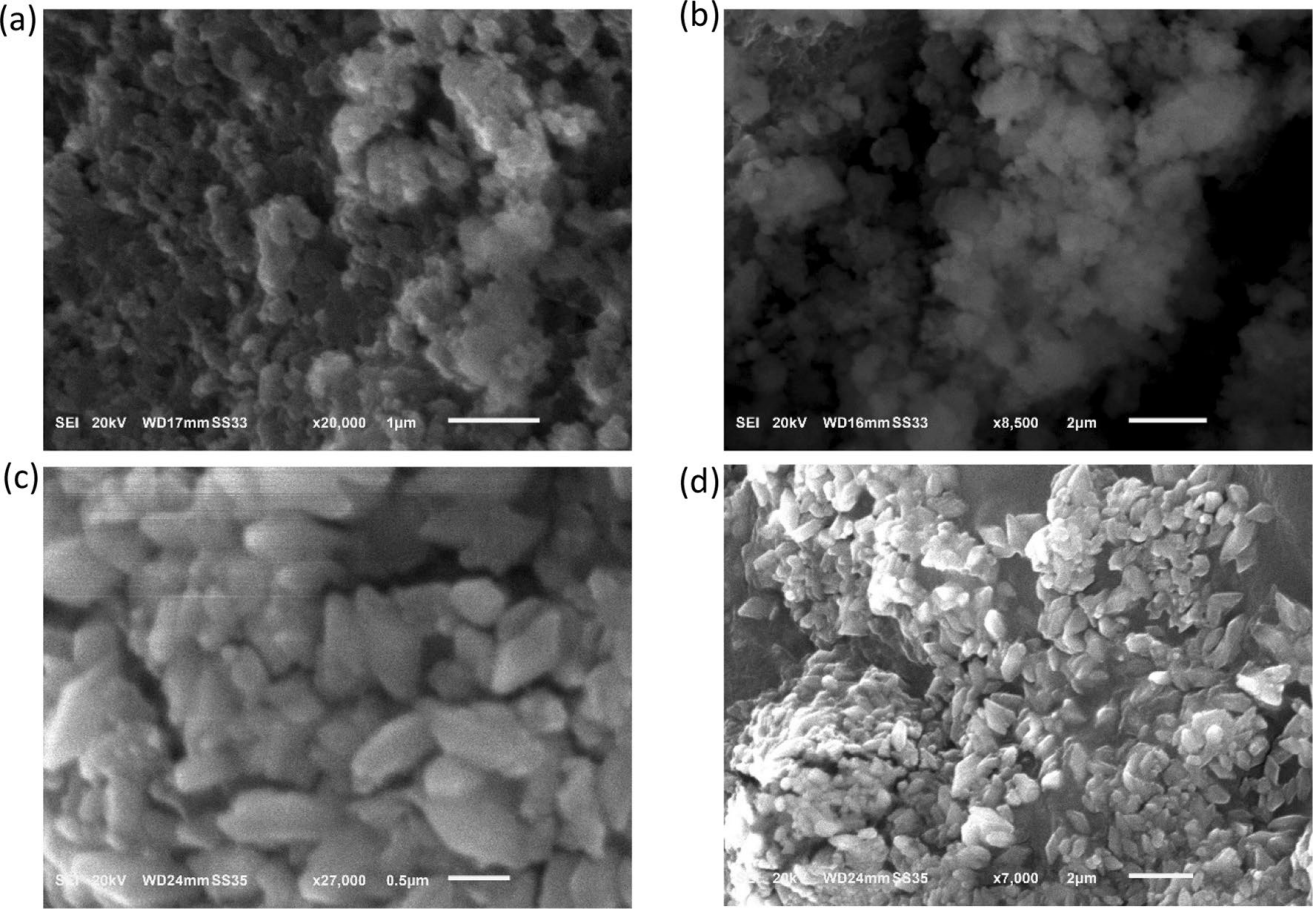
SEM micrographs of (**a,b**) CuO NPS-1 and (**b,c**) CuO NPs-2 at different magnifications.

The SEM micrographs of the CuO NPs-1 (synthesized using the extract of mint leaves) and CuO NPs-2 (synthesized using the extract of orange peels) are illustrated in Fig. 1. Figure 1a,b shows that the prepared CuO NPs-1 were mostly spherical in shape, while CuO NPs-2 appear with more aggregates (Fig. 1c,d). This can be due to the coating of different surface functional groups from the prepared extracts (see Fig. 3b). The same issue was observed with Khani et al.^33^. The SEM micrographs revealed that the synthesized CuO NPs were in the nanometer range of ~ 150 nm. Sankar et al.^37^ found that the size of CuO NP was 140 nm when it is synthesized with the extract of *Carica papaya* leaves. On the other hand, Prasad et al.^38^ obtained spherical CuO NPs with sizes of 40–70 nm when the leaves extract of *Saraca indica* was utilized.

The BET surface area of the synthesized CuO NPs were found ~20 m^2^ g^−1^. In literature, the prepared CuO NPs with a precipitation technique has surface area of 34 m^2^ g^−1^ with a size of 196 nm^39^. Another study found that the BET surface area of the prepared CuO NPs with the same technique was 1.7 m^2^ g^−1^ with a size of 140 and 180 nm^40^. On the other hand, Dörner et al.^41^ found the BET of sol–gel synthesized CuO NPs was 16 m^2^ g^−1^ with a size of 100–140 nm.

**Figure 2:**
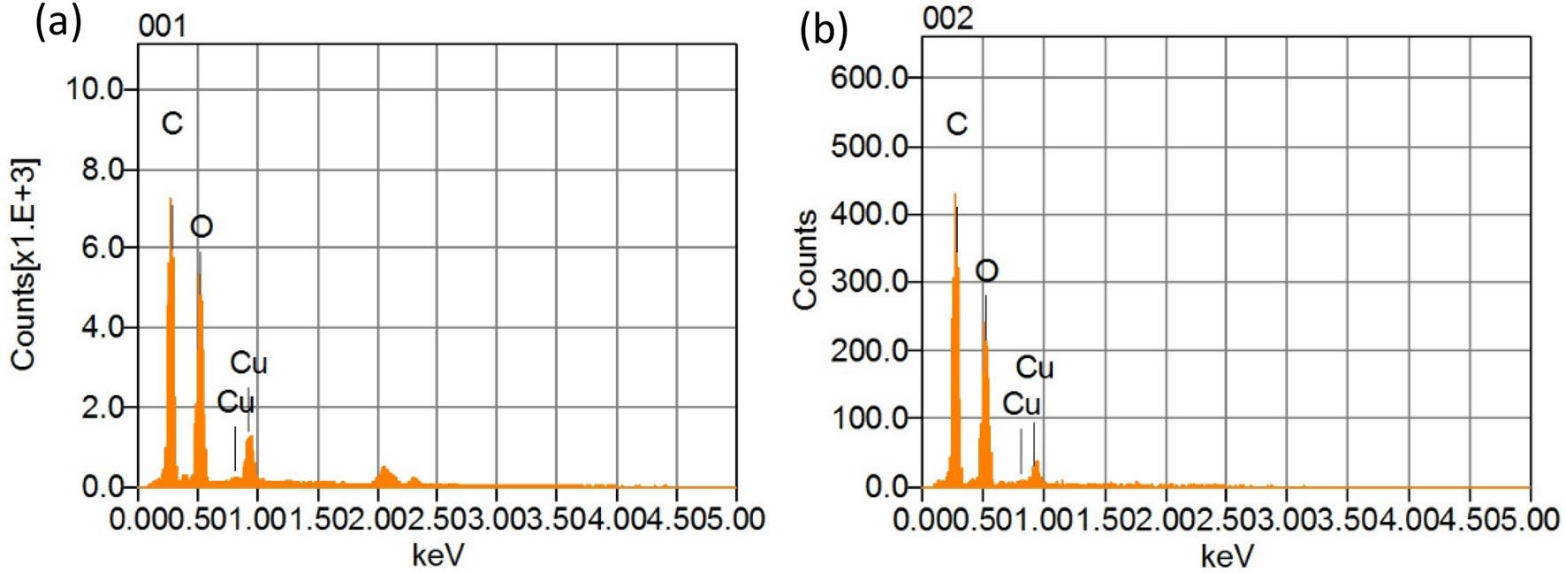
EDX spectra of (**a**) CuO NPs-1 and (**b**) CuO NPs-2.

The elemental compositions of the prepared CuO NPs were confirmed using Energy-dispersive X-ray (EDX) and the peaks obtained are illustrated in Fig. 2a,b. It is observed that the prepared CuO NPs are mainly composed of Cu, O and C without any trace of other materials. In both samples, EDX patterns show a strong signal peak at 1.0 keV representing Cu atoms. The detected carbon and high oxide peaks must be due to the phytochemicals already present in both plant extracts which are added in large volume. There are no other elements were detected even from the extract. The same findings were found with using the leaf extract of *Psidium guajava* as a reducing agent for the synthesis of CuO NPs^32^.

**Figure 3:**
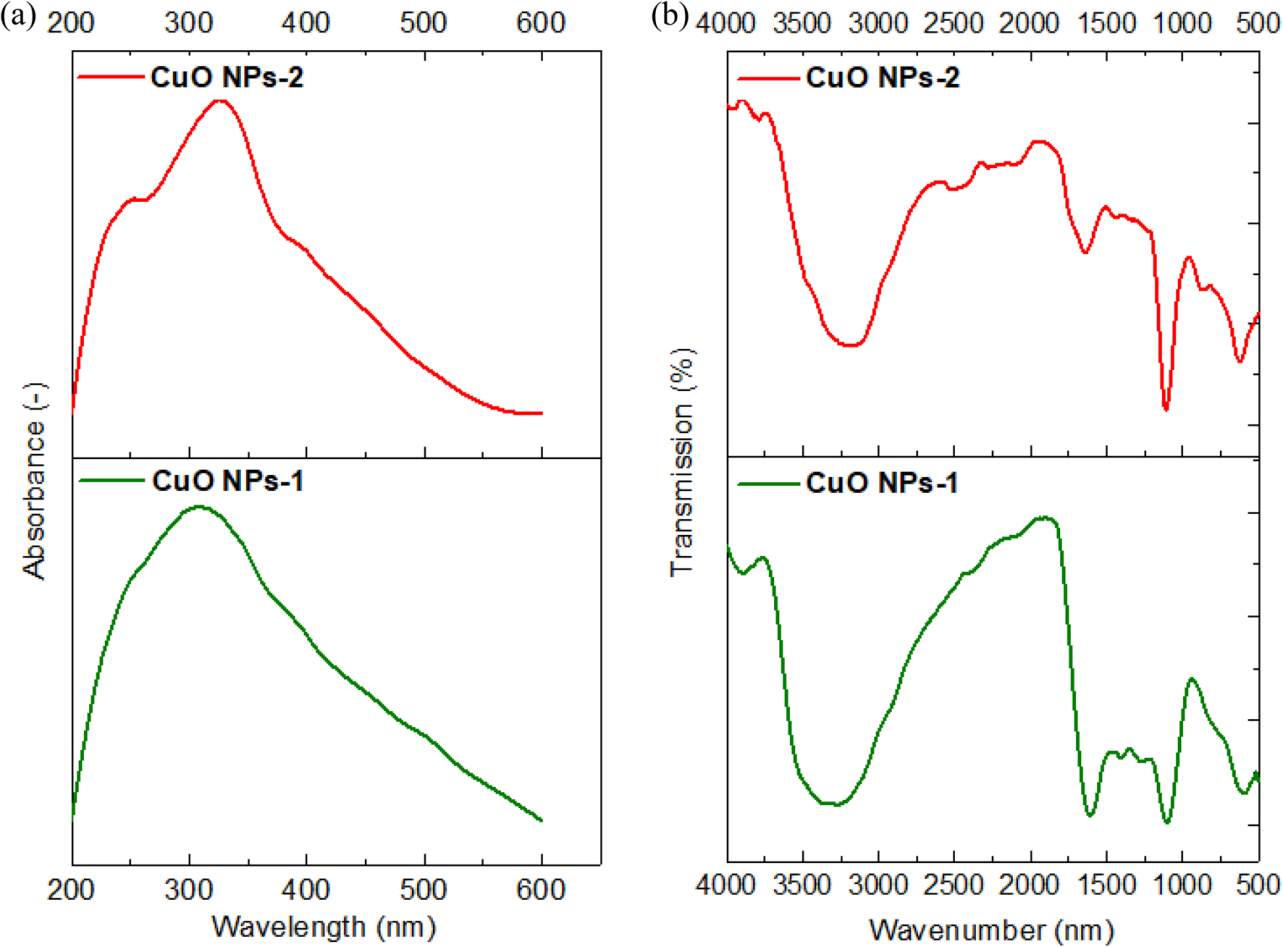
(**a**) UV–Vis spectra and (**b**) FT-IR Spectra of CuO NPs using mint extract (CuO NPs-1) and orange peel extract (CuO NPs-2).

The phytochemicals in the extracts are responsible for the formation of complexes with the copper salt that is reduced the ions to form nanoparticles. Hence, we observed the color transformation in the prepared Solutions and UV–Vis spectroscopy is used in the range of 200‒600 nm. Figure 3a indicates a noticeable peak at 325 nm due to the inter band transition of the core electrons of the CuO NPs. Aziz et al.^42^ used also mint leaf extract for the synthesis of CuO NPs and detected its absorption peak at 346 nm. Sankar et al.^37^ detected a strong absorbance peak between 250 and 300 nm suggesting the formation of CuO NPs.

The result of FT- IR spectrum of synthesized CuO NPs using the extract of mint and orange leaves were shown in Fig. 3b. The broad peaks at 3290 cm^−1^ correspond to the O–H stretching of the Phenols and alcohols. The most intense bands between 1603 and 1627 cm^−1^ represent C=O stretching band. The peak value at 1273 cm^−1^ shows the presence of C–O stretching of alcohols. The peak at 1100 cm^−1^ stands for C–N stretching aliphatic amines, or 1150–1085 cm^−1^ for strong C–O stretching aliphatic ether. The absorption at 1743 cm^−1^ was caused by C=O stretching esters, saturated aliphatic, aldehydes. The peaks at ~ 590 cm^−1^ and ~ 610 cm^−1^ exhibit the CuO phase. Similar characteristic peaks were observed by Priya et al.^43^ with *Aerva lanata*-mediated CuO NPs at 580 cm^−1^ and 525 cm^−1^. This indicated that the synthetic method conditions reflect the CuO phase.

### Metal ions treatment experiments

#### Effect of nanosorbents dose

**Figure 4:**
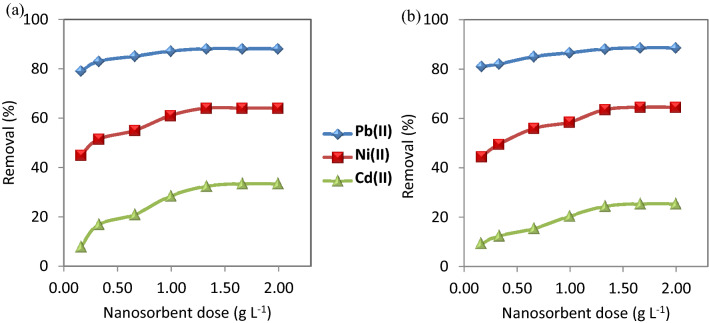
Effect of nanosorbent doses on the removal of the studied metal ions using (**a**) CuO NPs-1 and (**b**) CuO NPs-2 at initial concentration: 20 mg L^−1^, pH: 6, and contact time: 60 min.

The dose of nanosorbents has a great effect on the adsorption performance. Various dose concentrations (0.17, 0.33, 0.67, 1.00, 1.33, 1.67, and 2.00 g L^−1^) of CuO NP-1 and CuO NP-2 were used to evaluate the efficiency of removing the studied metal ions. Figure 4 Shows an increase in the removal efficiency of Pb(II), Ni(II), and Cd(II) with increasing in the dose of nanosorbents. The reason is due to the availability of more binding sites on the surface of the nanosorbents to the complexity of the metal ions. The selectivity sequence of CuO NPs for the adsorption process was Pb(II) > Ni(II) > Cd(II). Thus, the adsorption of Cd(II) is the least due to its lower electronegativity (1.69) and its bigger radius hydrated radius (0.404 nm) than Ni(II) (1.91 nm) and Pb(II) (0.401 nm).

Our results indicated that 0.33 g L^−1^ of CuO NPs can be used for further experiments because the nanosorbent dose is a key parameter in the cost analysis of the adsorption process. Therefore, it is recommended to use the lower nanosorbent dose but if have high adsorption performance. This nanosorbent dose is much less than one stated in literature. Sreekala et al.^44^ observed that the optimum dose of CuO NPs (synthesized with *Simarouba glauca* leaf extract) for 10 mg L^−1^ Pb(II) was 1.00 g L^−1^.

#### Effect of contact time

**Figure 5:**
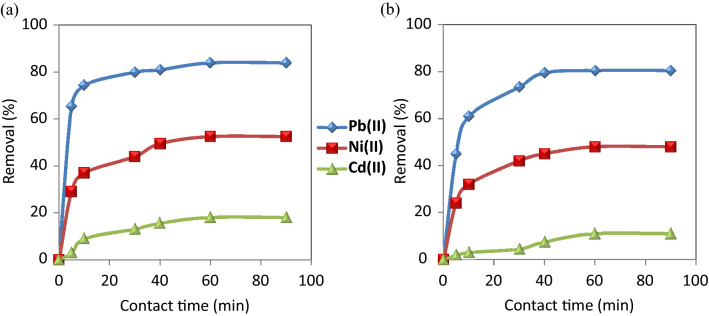
Effect of contact time on the removal of the studied metal ions using (**a**) CuO NPs-1 and (**b**) CuO NPs-2 at initial concentration: 20 mg L^−1^, pH: 6, and dose: 0.33 g L^−1^.

Figure 5 shows that the removal efficiency of the selected metal ions on CuO NPs required 60 min contact time to reach equilibrium. It is observed that the adsorption rate became almost fixed after 60 min and had a little effect on its rate. This can be attributed to the saturated capacity of the studied nanosorbents.

The removal efficiency of CuO NPs-1 was compared to CuO NPs-2 with the studied metal ions. The removal % of Cd (II), Ni(II) and Pb (II) were 18%, 52.5%, 84% and 11%, 48%, 80.5% when using CuO NPs-1 and CuO NPs-2, respectively. The variation in the removal % of heavy metals is due to the types of the used extracts and their volumes. They can influence the application of CuO NPs in the heavy metals removal. Due to the high intensity of the surface functional groups of CuO NPs-1 as detected in FT-IR (Fig. 3b), the highest percentage removal of the studied metal ions was obtained using CuO NPs-1. The reason for that can be the richness of mint leaves extract with various phytochemical constituents as reported in Alexa et al.^45^ and Thawkar^46^ compared to the orange peels extract^47^. Hence, we can conclude that CuO NPs-1 are effective in removing heavy metals.

#### Effect of pH

**Figure 6:**
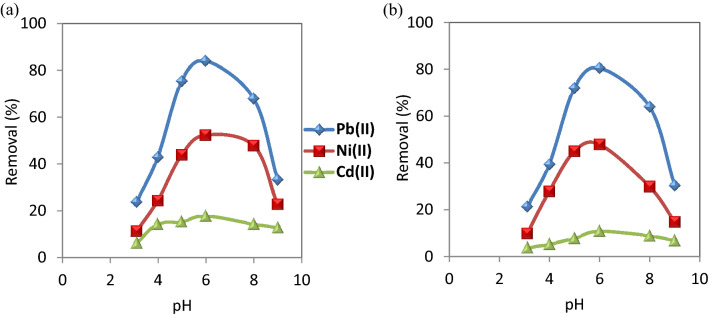
Effect of pH on the removal of the studied metal ions using (**a**) CuO NPs-1 and (**b**) CuO NPs-2 at initial concentration: 20 mg L^−1^, contact time: 60 min, pH: 6, and dose: 0.33 g L^−1^.

Removal of heavy metals from contaminated water depends largely on the pH of the solution. Consequently, the effect of pH on the adsorption of Pb(II), Ni(II),and Cd(II) on CuO NPs was evaluated with pH values, ranging from 3 to 9 at the equilibrium time. The results are shown in Fig. 6. When pH is increased from 3 to 6, the removal efficiency of Cd(II), Ni(II) and Pb(II) increased from 6.5, 11.5 and 24% to 18, 52.5 and 84%, respectively in the case of CuO NPs-1 which were higher than  those values of CuO NPs-2. Subsequent to these values,the adsorption rate decreased.

With increasing the pH values till 6, the removal % of the studied metal ions increased because of the decrement of the positive charge of the nanosorbent resulted in a lower electrostatic repulsion between the nanosorbents surface and metal cations as well as the competition decrement between the metal cations and protons of hydrogen ions for the functional groups of the nanosorbents^48^. In addition, pH values beyond 6 resulted in the precipitation of metal ions.

#### Effect of initial concentration of the selected metal ions

**Figure 7:**
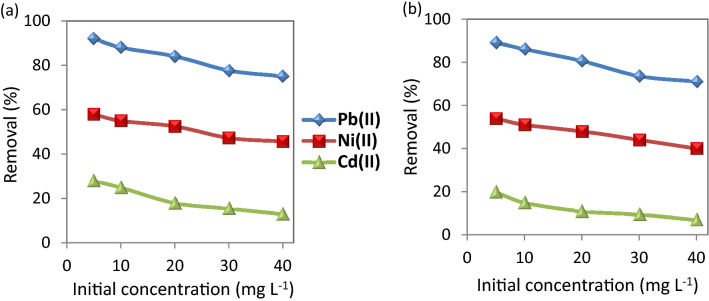
Effect of initial metal concentration on the removal of the studied metal ions using (**a**) CuO NPs-1 and (**b**) CuO NPs-2 at contact time: 60 min, and dose: 0.33 g L^−1^.

As shown in Figure 7, the adsorption of the metal ions at different concentrations indicates the removal % decreases with increasing the concentration of the metal ions. Utilizing CuO NP-1 nanosorbent in the removal of Pb(II), Ni(II), and Cd(II) that are ranging from 5 to 40 mg L^−1^ cause the removal % of Pb(II), Ni(II), and Cd(II) decreased from 92.0, 58.0, and 28.0% to 74.0, 45.7, and 13.0%, respectively. While the nanosorbent of CuO NP-2 led to the decrement of Pb(II), Ni(II), and Cd(II) removal from 89.0, 54.0, and 20.0% to 71.0, 40.0, and 7.0%, respectively. Similar findings were reported with CuO NPs in Jain et al.^36^ and Singh et al.^32^. This phenomenon illustrates a significant relationship between the adsorption efficiency and the metal concentration. At low metal concentrations, more absorbable vacant sites are available which lead to an increase in the prevalence of metal ions on the nanosorbent. At a higher concentration of metal ions, the available adsorbed sites become less and thus the removal rate of these ions decreases^23^.

**Figure 8:**
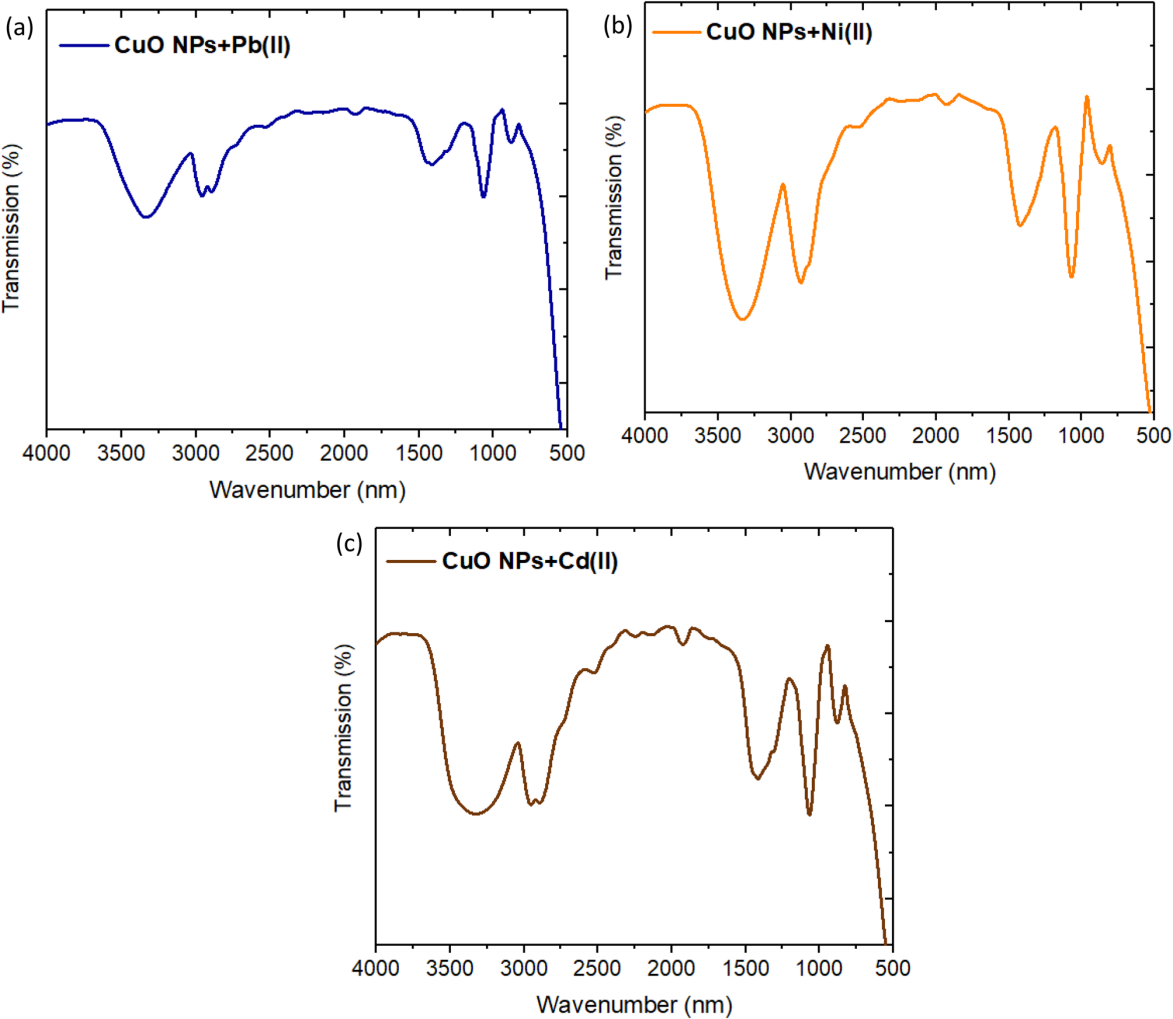
FT-IR Spectra of CuO NPs after adsorption of (**a**) Pb(II), (**b**) Ni(II), and (**c**) Cd(II).

In this work, the understanding mechanism of the studied metal ions removal could be predicted using FT-IR for the spent CuO NPs. After the metal ions adsorption, the O–H stretching bands get weaker and we observed new peaks as well as shifts in the intensities and positions of FT-IR bands as shown in Fig. 8. The band of CH_2_ and CH_3_ groups appeared which might be induced by C–H stretching vibration of CH_2_ and CH_3_ groups^49^. It became more intense and shifted with each metal ions removal. This band of CuO NPs is shifted to 2960, 2928, and 2956 cm^−1^ after removal of Pb(II), Ni(II), and Cd(II), respectively. Other shifts were observed in the bands of C–O stretching aliphatic ether to be 1063, 1068, 1070 cm^−1^ after removal of Pb(II), Ni(II), and Cd(II), respectively. The intense peaks appeared in the range of 1424–1416 cm^−1^ are attributed to –C–OH deformation. This indicates that the functional groups present on the synthesized CuO NPs were involved in the adsorption process of the studied metal ions.

### Adsorption models

#### Adsorption isotherms

Two isotherm models (Langmuir and Freundlich) were applied to describe the adsorption process. Their linear equations are expressed in Eqs. (3) and (4), respectively. The differentiation between the two models is that the Langmuir model suggests homogeneity of the surface of the nanosorbent and no further adsorption occurs once the available adsorption sites are filled, while the Freundlich model proposes heterogeneous of the surface of the nanosorbent.

**Figure 9:**
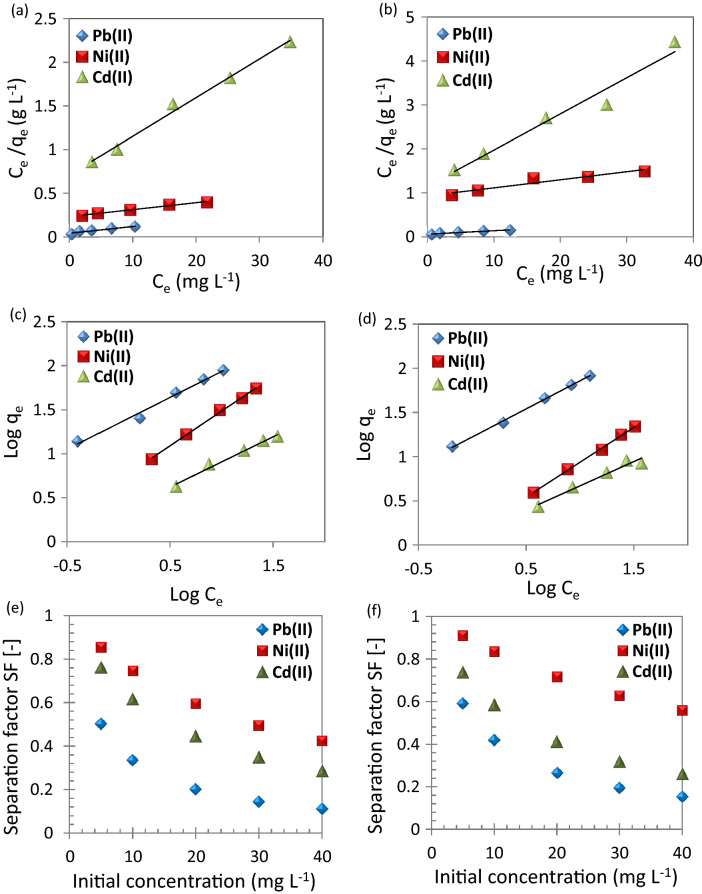
(**a,b**) Langmuir plots, (**c,d**) Freundlich plots, and (**e,f**) separation factor for the adsorption of metal ions; Pb(II), Ni(II), and Cd(II) using (**a,c,e**) CuO NPs-1 and (**b,d,f**) CuO NPs-2 at contact time: 60 min, dose: 0.33 g L^−1^, and pH: 6.

As shown in Figure 9, R^2^ values of the Freundlich models are higher than the Langmuir models. This indicates that the adsorption of metal ions to the surface of the CuO NPs is carried out by multiple, heterogeneous layers of the nanosorbent surface. Therefore, the adsorption of metal ions using CuO NPs can be described by the Freundlich model. On the other hand, the chemically synthesized CuO NPs (precipitation method) showed monolayer adsorption with Ni(II)^36^.

Table 1 illustrates the isotherm parameters for the adsorption of Pb(II), Ni(II), and Cd(II). The adsorption intensity (n) indicates the sorption driving force’s magnitude. n values are usually in the range of 0–1. The calculations of n values indicated that the adsorption isotherm is favorable because their values are < 1. The adsorption intensity can be also checked using separation factor (SF; Eq. (5)). Their values confirmed the findings of n values as illustrated in Fig. 9e,f. Furthermore, the SF values of CuO NP-1 were less than CuO NP-2 so the CuO NP-1 adsorption of the studied metal ions is expected to be more as confirmed from the experimental work. Desta^50^ found the adsorption of Cr(VI), Cd(II), Pb(II), Ni(II), and Cu(II) is favorable using teff straw waste due to the SF values were in the range of 0.298 to 0.986.

**Table 1:** Isotherm parameters and correlation coefficients for the investigated heavy metals using nano-sorbents (CuO NPs-1 and CuO NPs-2).

Heavy metals	Nano-sorbents					
	CuO NPs-1			CuO NPs-2		
	Pb(II)	Ni(II)	Cd(II)	Pb(II)	Ni(II)	Cd(II)
q_m exp_ (mg g^−1^)	88.80	54.90	15.60	82.80	21.90	8.4
**Langmuir model**						
q_m cal_ (mg g^−1^)	126.58	126.58	22.57	125.00	54.35	12.15
K_L_ (L mg^−1^)	0.19	0.03	0.06	0.14	0.02	0.07
R^2^	0.92	0.98	0.99	0.94	0.92	0.96
**Freundlich model**						
K_f_ (mg g^−1^) (mg L^−1^)^n^	22.15	4.98	2.16	16.71	1.41	1.33
n	0.59	0.79	0.57	0.64	0.79	0.55
R^2^	0.99	0.99	0.98	0.99	0.99	0.97

The maximum uptake capacity, q_m_ of Pb(II) was 88.80 and 82.80 mg g^−1^ using CuO NP-1 and CuO NP-2, respectively. Faisal et al.^22^ estimated the q_m_ of Pb(II) using sludge to be 20.41 mg g^−1^ under similar our experimental conditions except for the dose of 6 g L^−1^. The Langmuir affinity constant (K_L_) of CuO NPs-1 and CuO NPs-2 for Pb(II) adsorption was higher than K_L_ of Ni(II) and Cd(II). The high K_L_ value estimates the studied metal ions affinity to the available adsorption sites of CuO NPs. Such findings are attributed to the behavior of Pb(II) in the aqueous solutions. For instance, the high electronegativity of Pb(II) which is 2.10 and its small radius hydrated radius 0.401 nm^51^.

#### Adsorption kinetics

**Figure 10:**
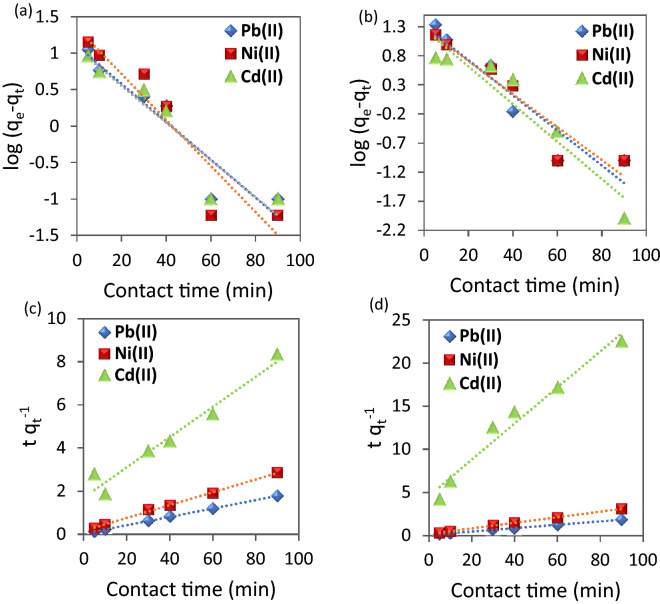
(**a,b**) Pseudo-first order and (**c,d**) Pseudo-second order for the adsorption metal ions using (**a,c**) CuO NPs-1 and (**b,d**) CuO NPs-2 at initial concentration: 20 mg L^−1^, dose: 0.01 g, and pH 6.

Figure 10a,b provides a straight line with slope (K_1_; min^−1^) and intercept equal to log q_e_. It is worth noting that the values of q_e exp_ are different from the calculated ones obtained from the pseudo-first order which indicates that this model is not valid to represent the adsorption process. On the other hand, Figure 10c,d represents linear plots of (t/q_t_) versus time*.* Its linear fit gives a straight line with slope of the rate constant (1/q_e_) and intercept 1/k_2_ q_e_^2^.

The highest correlation coefficients (R^2^) were obtained for pseudo-second order kinetic models (Table 2). The validation of pseudo-second order indicates that the adsorption capacity is related to the available active sites on nanosorbents^23^. Farghali et al.^52^ found the same behavior for Pb(II) kinetics removal by CuO NPs which assume the adsorption process is rate limiting process. However, they estimated the optimum contact time is 240 min for using CuO NPs synthesized from microwave radiation which is more than our reported optimum contact time (60 min). Both models’ parameters are summarized in Table 2. In addition, the values of initial adsorption rate (h; Eq. (8)) indicated that Pb(II) possesses the high rate compared to Ni(II) and Cd(II).

**Table 2:** Kinetic parameters and correlation coefficients for the investigated heavy metals using nano-sorbents (CuO NPs-1 and CuO NPs-2).

Heavy metals	Nano-sorbents					
	CuO NPs-1			CuO NPs-2		
	Pb(II)	Ni(II)	Cd(II)	Pb(II)	Ni(II)	Cd(II)
q_e exp_ (mg g^−1^)	50.50	31.56	10.90	48.40	12.10	6.91
**Pseudo-first order**						
q_e cal_ (mg g^−1^)	3.00	3.84	2.89	3.77	3.58	3.52
K_1_ (min^−1^)	0.06	0.07	0.05	0.07	0.06	0.07
R^2^	0.89	0.88	0.89	0.89	0.90	0.89
**Pseudo-second order**						
q_e cal_ (mg g^−1^)	51.28	33.56	14.26	50.76	30.96	4.77
K_2_ (g mg^−1^ min^−1^)	0.011	0.005	0.003	0.005	0.005	0.010
h (mg g^−1^ min^−1^)	29.76	6.14	0.59	13.09	5.21	0.22
R^2^	0.99	0.99	0.96	0.99	0.99	0.97

## Conclusions

The preparation of green CuO NPs was successful with the mint leaves and orange peels extracts as reducing agents. This proposed method holds several merits such as easy preparation, cost-effective, and safe compared to the chemical methods as well as the green synthesis method could be applicable for preparing other metal oxide nanoparticles. The EDX and UV–Vis spectroscopy confirmed the preparation of CuO NPs using both extracts. The adsorption application of CuO NPs on the removal of Pb(II), Ni(II), and Cd(II) is found to be dependent on the nanosorbent dose, the metal ions concentration, pH and the contact time. The optimum equilibrium contact time (60 min) and nanosorbent dose (0.33 g L^−1^) are less than those stated in literature for the adsorption of the studied metal ions. The affinity of these metal ions to CuO NPs followed the sequence Pb^2+^ > Ni^2+^ > Cd^2+^.

The optimum removal efficiency of Pb(II), Ni(II), and Cd(II) were found 84.00, 52.50%, and 18.00%, respectively and obtained at pH 6 for simulating wastewater under normal environmental conditions. The experimental data indicated that the Freundlich isotherm model fitted to the adsorption process as well as pseudo-second order. The maximum adsorption uptakes were 88.80, 54.90, and 15.60 mg g^−1^ for Pb(II), Ni(II), and Cd(II) with CuO NPs-1. These findings revealed that CuO NPs can be a good nanosorbent to purify water contaminated with heavy metals and its regeneration and reusing should be studied in the future. Furthermore, the environmental application performance of metallic oxide nanoparticles relies on the type of the used extract and its volume for the green synthesis method that influence the morphological properties of the produced nanoparticles and reflect its application performance.

## Materials and methods

### Preparation of plant extracts

Orange peels and mint leaves were collected from a local vegetable market. Then, we prepared the orange peel extract and mint leaves extract by washing them with double distilled water and drying at room temperature for 48 h. Each one was grinded, and we added 25 g in a standard beaker filled with 500 mL of double distilled water, the solution is boiled for 5 min (Fig. 10). Subsequent to boiling and leaving to the solution to cool down, we filtered and stored each extract at 4 °C and used it within a week as a reducing agent for preparing CuO NPs.

### Preparation of CuO NPs

**Figure 11:**
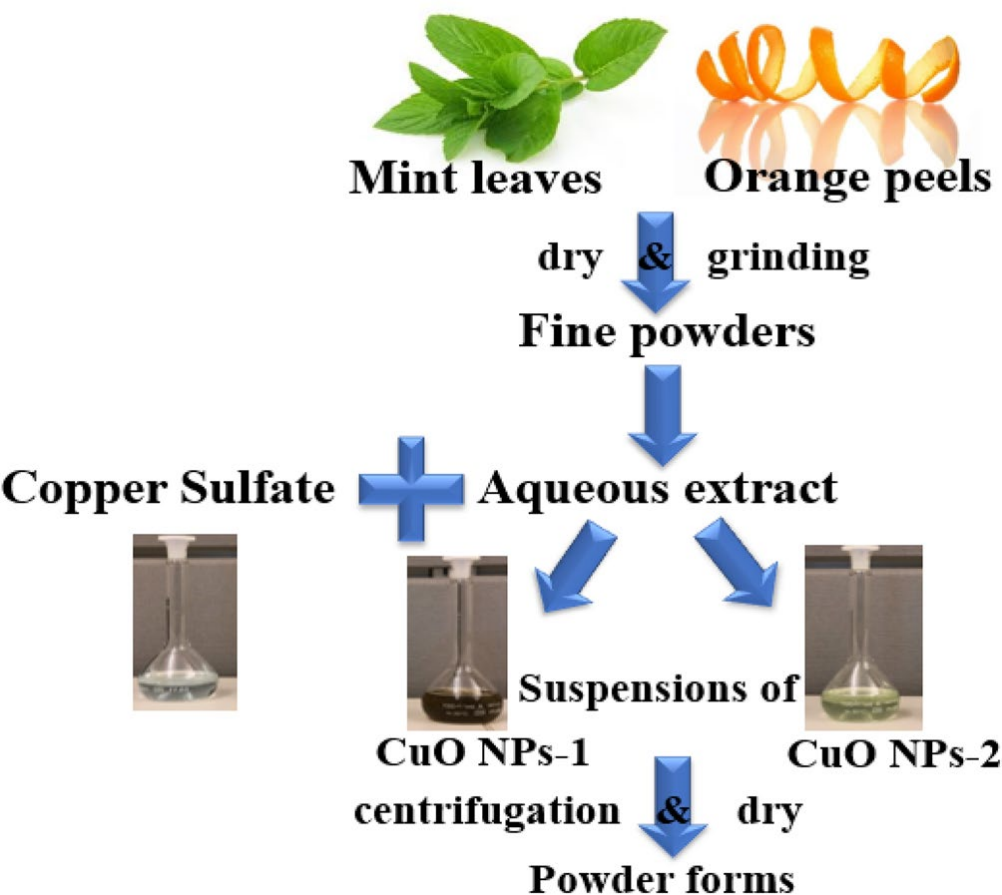
Schematic diagram for the green synthesis of CuO NPs.

In the green synthesis technique, 20 mL of either orange peel extract or mint leaves extract were added to 80 mL of copper sulfate (CuSO_4_) at a concentration of 0.01 M in a 250 mL Erlenmeyer flask and placed on a magnetic stirrer and heated to 50 °C, then the extract is slowly added to the solution for 20 min and then left stirred for 2 h where the solution changes when adding the mint leaves extract to the brown color,  the obtained nanoparticles denoted as CuO NPs-1, and when adding the orange peel extract, the color changed to the light green color, and the obtained nanoparticles denoted as CuO NPs-2. The two mixtures were left for 24 h at room temperature then separated using a centrifuge (12,000×g cycles) for 15 min and the nanoparticles were obtained after drying in an oven at 45 °C for 24 h. The schematic diagram for both nanoparticles synthesis is shown in Fig. 11.

### Detection and characterization of CuO NPs

The primary detections of CuO NPs were carried out by visual observation of the change in the color of the precursor. The synthesized nanostructures have been characterized by UV–Vis spectroscopy using Shimadzu UV-1700, Japan. The BET surface area of CuO NPs were measured with a Belsorp-miniX (Germany). Prior to this measurement, the samples were degassed at 140 °C. Scanning Electron Microscopy (SEM) coupled with Energy dispersive X-ray (EDX) was used to examine the surface morphology and size of the synthesized CuO NPs as well as its elemental composition. Fourier transform infrared spectroscopy (FT-IR) spectroscopy is used to identify the stretching and bending frequencies of molecular functional groups attached to CuO NPs surface^49^. Its spectra record was conducted in the range of 500–4000 cm^−1^.

### Metal ions treatment experiments

We have prepared artificial wastewater containing lead, nickel, and cadmium. Several factors were studied. For instance, the doses of CuO NPs-1 and CuO NPs-2 were 0.17, 0.33, 0.67, 1.00, 1.33, 1.67, and 2.00 g L^−1^. The optimum dose was fixed at 0.33 g L^−1^ when studying the other factors. The second factor was contact time at different times (5–90 min) and the time was fixed at 60 min when studying other factors. The third factor was studying the effect of the different metal concentrations (5–40 mg L^−1^) and the concentration was fixed at 20 mg L^−1^. The fourth factor was the pH of the solutions. It was adjusted in the range of 3–9 by 0.1 M NaOH or 0.1 M HCl and the pH was fixed at 6.00 when studying the other factors.

The experimental experiments were carried out by shaking 0.33 g L^−1^ of either CuO NPs-1 or CuO NPs-2 in 30 mL solution of each metal ions, with concentration range from 5 to 40 mg L^−1^, onto a bath shaker at 120 rpm. The adsorption capacity and removal percentage of the nanosorbents can be estimated with the following equations ^53–55^.

1 $${\text{q}}_{{\text{e}}} = \frac{{{\text{C}}_{{\text{o}}} - {\text{C}}_{{\text{e}}} }}{{\text{W}}} \times V,$$

2 $$\% R = \frac{{{\text{C}}_{{\text{o}}} - {\text{C}}_{{\text{f}}} }}{{{\text{C}}_{{\text{o}}} }} \times 100$$

where q_e_ is the equilibrium adsorption capacity (mg g^−1^), C_o_ is the metal ion initial concentration (mg L^−1^), C_e_ is the metal ion concentration (mg L^−1^) at equilibrium, V is the volume of solution (mL) and W is the weight (g) of nanosorbent, R is the removal percentage of the studied metal ions.

Isotherm and kinetics models are investigated to get the optimum conditions of the batch adsorption process. Langmuir and Freundlich models were used as they are most used isotherm models and can be compared to literature based on Eqs. (3) and (4). In addition, separation factor (SF; Eq. (5)) is calculated at different initial metal ion concentrations to express the adsorption process feasibility^23,56^.

3 $${\text{C}}_{{\text{e}}} /{\text{q}}_{{\text{e}}} = { 1}/{\text{K}}_{{\text{L}}} {\text{q}}_{{{\text{max}}}} + {\text{ C}}_{{\text{e}}} /{\text{q}}_{{{\text{max}}}} ,$$

4 $${\text{Log}}\left( {{\text{q}}_{{\text{e}}} } \right) \, = {\text{ log}}\left( {{\text{K}}_{{\text{f}}} } \right) \, + {\text{ n log}}\left( {{\text{C}}_{{\text{e}}} } \right) ,$$

5 $${\text{SF }} = { 1}/\left( {{1} + {\text{K}}_{{\text{L}}} {\text{C}}_{0} } \right),$$

where K_L_ is the Langmuir adsorption equilibrium constant related to the affinity between the metal ions and nanosorbents (L mg^−1^), n is the measure of adsorption intensity and it indicates the relative distribution of energy sites. K_f_ (mg g^−1^) (L mg^−1^)^n^ constant is concerned with the ability of nanosorbent to adsorb. SF is the separation factor (dimensionless).

The kinetics removal of the studied metal ions can be explained using pseudo-first order (Eq. (6)) and pseudo-second order (Eq. (7)). Initial adsorption rate (h) is calculated using Eq. (8).

6 $${\text{Log }}\left( {{\text{q}}_{{\text{e}}} - {\text{ q}}_{{\text{t}}} } \right) \, = {\text{ log q}}_{{\text{e}}} {-} \, \left( {{\text{K}}_{{1}} /{2}.{3}0{3}} \right){\text{ t}},$$

7 $${\text{t}}/{\text{q}}_{{\text{t}}} = { 1}/{\text{K}}_{{2}} {\text{q}}_{{\text{e}}}^{{2}} + \, \left( {{1}/{\text{q}}_{{\text{e}}} } \right){\text{ t}},$$

8 $$h = K_{2} q_{e}^{2} ,$$

where q_t_ is adsorption capacity at contact time (t), K_1_ is the pseudo first order rate constant (min^−1^), K_2_ the pseudo second order rate constant (g mg^−1^ min^−1^).
